# Comparative Study of Raw and Boiled Silver Pomfret Fish from Coastal Area and Retail Market in Relation to Trace Metals and Proximate Composition

**DOI:** 10.1155/2014/826139

**Published:** 2014-08-12

**Authors:** Roksana Huque, M. Kamruzzaman Munshi, Afifa Khatun, Mahfuza Islam, Afzal Hossain, Arzina Hossain, Shirin Akter, Jamiul Kabir, Yeasmin Nahar Jolly, Ashraful Islam

**Affiliations:** ^1^Food Technology Division, Institute of Food and Radiation Biology, Atomic Energy Research Establishment, GPO Box No. 3787, Dhaka 1000, Bangladesh; ^2^Chemistry Division, Atomic Energy Centre, 4 Kazi Nazrul Islam Road, GPO Box No. 164, Ramna, Dhaka 1000, Bangladesh

## Abstract

Trace metals concentration and proximate composition of raw and boiled silver pomfret (*Pampus argenteus*) from coastal area and retail market were determined to gain the knowledge of the risk and benefits associated with indiscriminate consumption of marine fishes. The effects of cooking (boiling) on trace metal and proximate composition of silver pomfret fish were also investigated. Trace element results were determined by the Energy Dispersive X-ray Fluorescence (EDXRF) Spectrometer wherein fish samples from both areas exceeded the standard limits set by FAO/WHO for manganese, lead, cadmiumm and chromium and boiling has no significant effects on these three metal concentrations. Long-term intake of these contaminated fish samples can pose a health risk to humans who consume them.

## 1. Introduction

Fish is a healthy food for most of the world's population particularly developing countries in contrast to meat, poultry, and eggs. Fish provides comparatively cheap and readily available protein sources (about 15 to 20 percent) in addition to long chains of n-3 fatty acids, amino acids, vitamins, and minerals which contributes to healthier nutritional options for a balance dietary intake [1, 2]. Among the all fishes, marine fish are very rich sources of protein and various mineral components. The total content of minerals in raw flesh of marine fish is in the range of 0.6–1.5% of wet weight [3].

Trace metals are present in water from natural sources such as the rocks of the sea bed and also accumulated as a result of human activities such as emissions from industrial processes. These elements are taken up by marine fishes which are higher up the food chain. As a result, the concentrations of many elements including mercury, arsenic, lead, and cadmium in fish can be relatively high compared to other foods. Many of these metals such as iron, copper, cobalt, manganese, molybdenum, nickel, and zinc are essential trace elements and play important roles in biological systems. Meanwhile, mercury, lead, and cadmium are toxic, even in trace amounts [4]. Moreover, elevated concentration of manganese and nickel has been found to be toxic to aquatic organism [5, 6].

To monitor trace metals concentrations in the coastal environment, marine fishes have been widely used as bioindicators due to their wide range of distribution. Several studies have been carried out on metal pollution in different species of edible fish. Predominantly, fish toxicological and environmental studies have prompted interest in the determination of toxic elements in seafood [7–10]. According to the Codex Committee for Food Additives and Contaminants, dietary intakes of heavy metals with high public concern need to be monitored on a regular basis and rapidly updated to identify recent dietary intakes of heavy metals in developing countries. Bangladesh, as one of the developing countries, definitely needs a monitoring system to ensure a safe food supply, especially because the average diet includes an appreciable amount of fish, which typically contain high levels of heavy metals. The silver pomfret,* Pampus argenteus* is one of the most valuable and demanded fishes due to its high market value for export. Thus the safety of this fish has been a growing interest to determine the levels of contaminants to minimize the potential health risk to humans who consume them. The purpose of the present study was to determine the concentrations of trace metals by using Energy Dispersive X-ray Fluorescence (EDXRF) Spectrometer along with proximate composition of silver pomfret fish from costal area of Cox's Bazar and retail market of Dhaka city where silver pomfret fish were supplied from coastal area of Bay of Bengal. To evaluate the possible effect of cooking (boiling) on the concentration of proximate composition, trace metals, the concentrations obtained in the cooked products were compared with the concentrations found in the same raw fish. It is expected that the results of this research will assist in acquiring information about the level of toxic metals in this commercially important fish.

## 2. Materials and Methods

### 2.1. Sampling Sites

**Figure 1:**
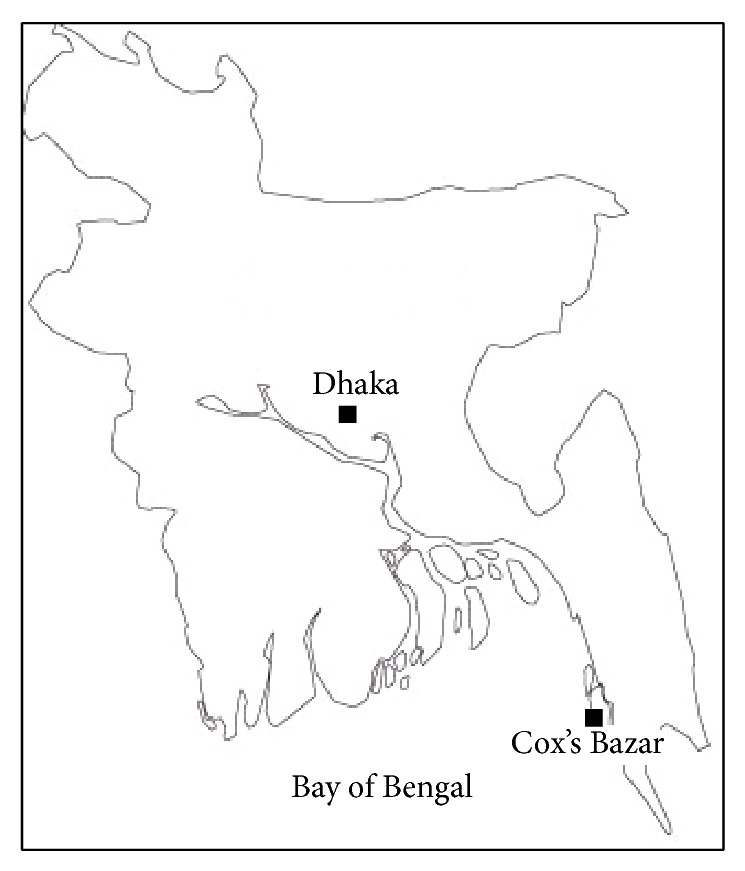
Sample collection sites.

To measure the bioavailability of trace metals and other physicochemical compositions, fresh silver promfret fishes were collected directly from local fishermen of Cox's Bazar located at 23.30°–21.56°N, 91.50°–92.23°E in Southeastern Bangladesh along the Bay of Bengal. Sample is also collected from retail market in Dhaka City (Figure 1).

### 2.2. Analytical Method by EDXRF

Energy Dispersive X-ray Fluorescence (EDXRF) Spectrometer (Model: Epsilon 5) was used as major analytical technique for carrying out elemental analysis in fish samples. Details of the instrument are described elsewhere [11].

### 2.3. Sample Preparation for EDXRF Analysis

Fish samples collected from either way were taken to the laboratory, cleaned up by washing with tap water several times to remove any dirt, and kept in a freezer. Later on, the samples were washed with deionised water several times and dissected in such a way so that the whole body tissue might be taken to the analysis. The samples were then dried at 55°C to constant weight. Finally the dried samples were ground to fine powder using carbide mortar and pestle.

### 2.4. Sample Irradiation with X-ray Beam

For irradiation of the sample with X-ray beam, 2 g of each powdered fish samples was pressed into a pellet of 25 mm diameter with a pellet maker (Automatic Hydraulic Presses, model: 3889-4NEI) and loaded into the X-ray excitation chamber with the help of automatic sample changer system. The irradiation of all real samples was performed by assigning a time-based programme controlled by a software package provided with the system. The standard materials were also irradiated under similar experimental conditions for construction of the calibration curves for quantitative elemental determination in the respective samples. The generated X-ray spectra of the materials were stored into the computer.

### 2.5. Concentration Calibration

A direct comparison method based on EDXRF technique was used for elemental concentration measurement [12, 13]. In comparison method, standards are set to construct the calibration curves. Again key to this comparison method is that both the standard and the samples have to be of similar kinds, so that they can produce identical sensitivity and the matrix effects are nullified. Hence to comply with the fact, three fish standards (Tuna-1, Tuna-2, and Tuna-3) were used for the purpose to construct the calibration curves used to carry out elemental analysis in fish samples. The calibration curve constructed for each element was based on its K X-ray and L X-ray line sensitivity as a function of its atomic number. Finally to verify the accuracy of the curves, a standard reference material was checked through the analysis. The results obtained for elements of interest and the certified values for corresponding elements are shown in Table 1. All the results with respect to certified known values were found to be in agreement as the range of error did not go beyond acceptable limit.

### 2.6. Proximate Composition

The percentage of proximate composition of fish was determined by conventional method of Association of Official Analytical Chemists (AOAC) [14]. Triplicate determinations were carried out on each chemical analysis.

*Estimation of Moisture*. The initial weight of the sample was taken, and then samples were dried in an oven at about 105°C for about 5 to 6 h until constant weight was reached. The percentage of moisture content was determined.

*Protein Determination*. The protein content of the fish was determined by micro Kjeldahl method AOAC [14]. It involves the conversion of organic nitrogen to ammonium sulphate by digestion of fish flesh with concentrated sulphuric acid in a micro Kjeldahl flask. The digest was diluted, made alkaline with sodium hydroxide, and distilled. The liberated ammonia was collected in a boric acid solution and total nitrogen was determined titrimetrically. The percentage of protein in the sample was calculated. 

*Estimation of Fat*. For the estimation of fat content, the dried samples left after moisture determinations were finely ground and the fat was extracted with chloroform and methanol mixture AOAC [14]. After extraction, the solvent was evaporated and the extracted materials were weighed. The percentage of the fat content was calculated. 

*Estimation of Ash*. The ash content of a sample is residue left after ashing in a muffle furnace at about 550–600°C till the residue becomes white. The percentage of ash was calculated by subtracting the ash weight from initial weight.

### 2.7. Statistical Analysis

Statistical procedures were performed using SPSS for Microsoft version 18.0 software package (SPSS Chicago, IL) with five percent level of significance.

## 3. Results and Discussion

### 3.1. Trace Metal Concentrations in Raw and Cooked (Boiled) Fish

The concentrations of trace metals (Cu, Fe, Zn, Cd, Mn, Pb, As, and Cr) in the analysed raw and cooked (boiled) fishes are listed in Table 2. A detailed discussion of the health effect of the above-mentioned elements are presented below.

#### 3.1.1. Copper (Cu)

Cu (Copper) forms an integral part of several enzymes and it is necessary for the synthesis of haemoglobin. In the present study, mean Cu concentrations in fresh fish from costal area (Cox's Bazar) and retail market (Kawran Bazar) were found to be 3.33 and 3.99 mg/kg, respectively, which were less than permissible limit (10 mg/kg) set by FAO/WHO [15]. This result agreed with the result reported elsewhere in fish samples (Ruhi, Tilapia, and Catfish) of the river Yamuna, Delhi (0.9 mg/kg to 3.7 mg/kg) [16]. The present study showed that boiling and sampling site had no significant (*P* ≥ 0.05) effects on Cu concentrations.

#### 3.1.2. Iron (Fe)

Iron is a necessary metal for human body, which forms part of haemoglobin, allows oxygen to be carried from the lungs to the tissues. The Fe content of fresh fish from coastal area (98.02 mg/kg) was significantly higher than those of retail market (79.47 mg/kg). This might be due to frozen storage of fish at retail market. It has also been found that Fe content was significantly decreased in frozen Hamour fish [17]. The reported iron in the present study was higher than pomfret fish from southwest of Peninsula Malaysia (6.84 mg/kg) [4], Northeast coast of India (60 mg/kg), [18] and Karachi Harbour (6.751 mg/kg) [19] but lower than world standard (300 mg/kg) [15]. Boiling showed no marked difference in Fe content compared to raw fish in both cases. This phenomenon was also found in case of boiled striped snakehead fish [20].

#### 3.1.3. Zinc (Zn)

Higher Zn content (25.25 mg/kg) was found in raw silver pomfret fish from costal area of Cox's Bazar along the Bay of Bengal than those of retail market (24.40 mg/kg); frozen storage of fish might be responsible for this type of observation. Silver pomfret fish from Karachi Harbour (3.975 mg/kg) [19] and costal water in Eastern India along the bay of Bengal (34.1 mg/kg) [18] showed a lower Zn concentration. Zn is essential micronutrients that are a component of more than 300 enzymes needed to play a role in many biological functions in the human body [21]. However, Zn becomes poisonous when it exceeds its maximum value. The Zn content in the present study is lower than permissible limit (150 mg/kg) by FAO/WHO [15] and FDA recommended health-criteria concentrations of Zn (480 mg/kg) [22]. Similar to Fe content, Zn content also was found to be insignificant (*P* > 0.05) in boiled fish and similar results were found in striped snakehead fish [20]. Conversely, it was reported that decreased Zn content was noticed in boiled rainbow trout [23].

#### 3.1.4. Cadmium (Cd)

Mean concentration of cadmium in raw and cooked fish was found to be 6.02-6.03 mg/kg in both raw and boiled fish from costal and retail market, respectively, and ANOVA analysis showed that there is no significant difference between the levels of cadmium in raw and boiled silver pomfret fish samples from both sources. In a study, relatively lower (2.10 mg/kg) cadmium level has been found in silver pomfret fish from Indian coast of Bay of Bengal [24]. On the other hand, high concentration of cadmium (4.35–6.38 mg/kg) level was reported in demersal species* N*.* japonicas* [25]. However the level of cadmium in the present study was higher than the WHO/FAO [26] set limit (1.0 mg/kg) which is quite alarming since cadmium is known as a human carcinogen and its critical target is the kidney for general population [27]. Acute toxicity caused by Cd containing food is very unusual but chronic exposure may be frequent [28].

#### 3.1.5. Manganese (Mn)

Manganese is a metal with low toxicity but has a considerable biological significance and seems to accumulate in fish species. Level of Manganese in raw silver pomfret fish from Cox's Bazar coastal area and retail market were 6.93 and 6.29 mg/kg, respectively. Boiling had no significant effect on Mn level in fish from both areas as also insignificant difference in Mn concentration in striped snakehead fish after boiling was found [20]. The observed levels of Mn were higher than silver pomfret fish (3.0 mg/kg) from costal water in Eastern India along the Bay of Bengal [18] and also higher than standard limit (5.4 mg/kg) set by FAO/WHO [15].

#### 3.1.6. Lead (Pb)

A significantly higher Pb concentration was found in both raw (15.33 mg/kg) and boiled fish (15.34 mg/kg) from coastal area compared to those of retail market (Table 2). The outcomes of this study are extremely higher than the permissible limits (2 mg/kg) set by WHO [29]. Extremely higher lead concentration (98.5 *μ*g/g) has been found in pomfret fish from coastal area of West Bengal, India [30]. The high values of lead in the studied samples indicate that the aquatic environment of Bay of Bengal are highly stressed with respect to the fact that lead may be due to soil erosion and leaching gasoline combustion and municipal and industrial wastes and runoff [31]. Higher concentration of Pb indicated possible health risks associated with consumption of these fishes; hence Pb is a cumulative body poison, which can affect every organ and system in the body. Exposure to its high level can severely damage the brain and kidneys and ultimately cause death and long-term exposure results in anaemia, abdominal pain, arthritis, depression, thyroid imbalances, tooth decay, and so forth, [32].

#### 3.1.7. Arsenic (As)

Arsenic is a widely distributed metalloid, occurring in rock, soil, water, and air. Arsenic is regarded as human carcinogen from extremely low levels of exposure. Its low level exposure causes nausea and vomiting, decreased production of RBCs and WBCs, and abdominal pain and long-term exposure causes darkening of skin and appearance of small corns on palm soles [33]. The observed arsenic level in raw and boiled fish from coastal area was 4.32 and 4.89 mg/kg, respectively, and fish from retail market was 3.49 to 3.51 mg/kg, respectively. ANOVA analysis revealed that arsenic value was higher in raw fish from coastal area than those of retail market. Arsenic level in boiling fish was not significantly different with their respective raw fish from both collecting areas. It has been reported that arsenic level in silver pomfret fish from West Bengal Coast of Bay of Bengal was 0.57 mg/kg which was lower than the observed value [24]. The WHO/FAO recommended the maximum permissible levels for arsenic in sea fish is 5.00 mg/kg [34].

#### 3.1.8. Chromium (Cr)

Chromium is a mineral that humans require in trace amounts. Chromium (VI) compounds are toxic and known human carcinogens, whereas chromium (III) is an essential element. The chromium concentrations determined in raw fish from coastal area and retail market were 13.51 and 12.40 mg/kg, respectively. As previously reported, the concentration of chromium in silver pomfret from Karachi Harbour was 0.62 mg/kg, wet weight [19]. The changes in Cr concentration in boiled fish were found to be insignificant (*P* > 0.05) compared to their respective raw fish. The observed Cr levels were much higher than tolerable standards of 0.05 mg/kg and 0.491 *μ*g/g, which may be due to industrial processes [35–37].

### 3.2. Proximate Composition

Results of moisture content, total protein, total lipid, and total ash contents of the fresh and boiled muscle of pomfret fish from costal area and retail market are shown in Table 3.

The moisture content was not significantly different between raw fish from coastal area and retail market and was decreasing after boiling (Table 3). Similar results have been found in boiled rainbow trout fillets [38]. On the other hand, some researchers disagree with our findings in case of three commonly consumed marine fishes in Nigeria [39]. It was also found that the moisture content decreased in all methods of cooking except for the boiled fillets of striped snakehead fish [20].

Protein content ranges from 20 to 20.3% in raw fish from both areas. A significantly higher rate of total protein content was found in boiled fish. This might be due to reduction of moisture content. Increased total protein was also found with reduced rate of moisture content in boiled rainbow trout fillets [38].

In the present study, the difference of fat and ash content in fish muscle from retail market and coastal area was insignificant and fresh and boiled samples had relatively similar fat and ash content, which may be due to the fact that the temperature at which the boiling was done was not high enough to cause any morphological change in the fish samples. Similarly, boiling did not impart any significant change on the oil and ash contents of the marine fish samples [39].

## 4. Conclusion

The international official regulatory agencies like WHO and FAO have set limits for trace metal contaminations above which the fish and fishery products are unsuitable for human consumption. However, in Bangladesh there is no safety levels of trace metals in fish tissues although fishes are major part of the human diet in Bangladesh.

The present study revealed that the studied Cd, Cr, and Pb concentrations were high in the tissues of the studied fish compared to permissible limits which might pose acute toxicological risks to human health. The coastal and marine pollution might result from the crude oil transportation systems, water-oil from different sea cargo, ships, and mechanized vessels, workshop, refinery handling loss, dumping of ballast and bilge water, and so forth. In Bangladesh, more than 50% of the marine oil pollution comes from urban activities and thrown river runoff [40, 41]. Besides this, a number of accidental spillage or discharge of crude petroleum at the coast of Bay of Bengal, shrimp and fish farming, and recent offshore hydrocarbon drilling operation in the exclusive economic zone (EEZ) of Bangladesh could be a factor of marine pollution [42]. The average concentration of cadmium, lead, and chromium in the present study was above permissible limits. Long-term intake of contaminated fish samples could lead to toxicity of heavy metals in human beings. Therefore, consumption of such fishes should be monitored to avoid the adverse effects brought about by lead, cadmium, and chromium.

Finally, we recommended that the assessment of trace metals in marine fish, particularly, should be done regularly to prevent the ever-increasing population from being exposed to harmful levels of these elements. In addition, guidance of people and farmers of both agriculture and aquaculture, about the instruction for use of pesticides, chemicals, drugs, and control of house wastewater spreading in rivers and crops, is necessary.

## Figures and Tables

**Table 1:** Analysis of standard reference materials showing comparison between present results and the certified values (mg kg^−1^).

Elements	Tuna fish		
	Results obtained	Certified values	Error (%)
Mn	2181	2140	−1.92
Fe	39341	43200	8.93
Cu	3405	3420	0.44
Zn	4197	4180	−0.41
As	1420	1540	7.79
Pb	5443	5520	1.39

**Table 2:** Effects of sampling location and boiling on trace metal concentration (in mg/kg) in the silver pomfret fish muscle.

Trace metal (mg/kg)	Fish from coastal area mean ± SD		Fish from retail market mean ± SD		World standard mg/kg
	Raw	Boiled	Raw	Boiled	
Copper (Cu)	3.33 ± 1.19^a^	3.55 ± 1.22^a^	3.99 ± 0.42^ab^	5.56 ± 1.99^b^	10 (FAO/WHO, 1984)
Iron (Fe)	98.02 ± 2.21^a^	99.77 ± 4.05^a^	79.47 ± 2.15^b^	80.24 ± 3.46^b^	300 (FAO/WHO, 1984)
Zinc (Zn)	25.25 ± 0.29^a^	24.82 ± 0.18^bc^	24.4 ± 0.27^c^	24.49 ± 0.46^d^	150 (FAO/WHO, 1984)
Manganese (Mn)	6.93 ± 1.21^a^	6.29 ± 0.22^b^	6.39 ± 0.43^c^	6.21 ± 0.22^d^	5.4 (FAO/WHO, 1984)
Cadmium (Cd)	6.02 ± 1.09*E* − 15^a^	6.03 ± 0.01^b^	6.02 ± 0.003^c^	6.02 ± 0.003^d^	1 (FAO/WHO, 1984)
Lead (Pb)	15.33 ± 0.07^a^	15.34 ± 0.38^a^	14.58 ± 0.19^b^	14.38 ± 0.14^b^	2 (WHO, 1985)
Chromium (Cr)	13.51 ± 0.37^a^	14.59 ± 1.26^bac^	12.4 ± 0.57^ca^	12.75 ± 0.55^ca^	0.05 (FAO/WHO, 1990)
Arsenic (As)	4.32 ± 0.73^a^	4.89 ± 0.29^a^	3.49 ± 0.10^b^	3.51 ± 0.09^cb^	5 (FAO/WHO, 2004)

Each replicate contained 3 treatment units. Mean values with different superscript letters are significantly different at *P* = 0.05 and LSD values are at *P* = 0.05.

**Table 3:** Effects of sampling location and boiling on proximate composition in the silver pomfret fish muscle.

Proximate composition	Fish from coastal area mean ± SD		Fish from retail market mean ± SD	
	Raw	Boiled fish	Raw	Boiled
Moisture (%)	75.35 ± 0.43^a^	70.57 ± 0.95^bc^	75.87 ± 0.26^a^	71.38 ± 0.66^cb^
Protein (%)	20.01 ± 0.60^a^	22.01 ± 0.68^bc^	20.30 ± 0.74^a^	22.22 ± 0.62^cb^
Fat (%)	1.64 ± 0.13^a^	2.19 ± 0.63^a^	1.95 ± 0.08^a^	2.35 ± 0.46^a^
Ash (%)	2.48 ± 0.16^a^	2.39 ± 0.08^a^	2.52 ± 0.06^a^	2.4 ± 0.13^a^

Each replicate contained 3 treatment units. Mean values with different superscript letters are significantly different at *P* = 0.05 and LSD values are at *P* = 0.05.

## References

[1] Hajeb P., Jinap S., Ismail A., Fatimah A. B., Jamilah B., Abdul Rahim M. (2009). Assessment of mercury level in commonly consumed marine fishes in Malaysia. *Food Control*.

[2] Food and Agriculture Organization (FAO) Nutritional elements of fish. http://www.fao.org/fishery/topic/12319/en.

[3] Sikorski Z. E. (1990). *Resources Nutritional Composition and Preservation*.

[4] Nurnadia A. A., Azrina A., Amin I., Mohd Yunus A. S., Mohd Izuan Effendi H. (2013). Mineral contents of selected marine fish and shellfish from the west coast of Peninsular Malaysia. *International Food Research Journal*.

[5] Kempster P. L., Hattingh W. A. J., Van V. H. R. (1982). Summarized water quality criteria.

[6] Khangarot B. S., Ray P. K. (1987). Correlation between heavy metal acute toxicity values in *Daphnia magna* and fish. *Bulletin of Environmental Contamination and Toxicology*.

[7] Begum A., Harikrishna S., Khan I. (2009). Analysis of heavy metals in water, sediments and fish samples of Madivala Lakes of Bangalore, Karnataka. *International Journal of ChemTech Research*.

[8] Tabari S., Saravi S. S. S., Bandany G. A., Dehghan A., Shokrzadeh M. (2010). Heavy metals (Zn, Pb, Cd and Cr) in fish, water and sediments sampled form Southern Caspian Sea, Iran. *Toxicology and Industrial Health*.

[9] Kalogeropoulos N., Karavoltsos S., Sakellari A., Avramidou S., Dassenakis M., Scoullos M. (2012). Heavy metals in raw, fried and grilled Mediterranean finfish and shellfish. *Food and Chemical Toxicology*.

[10] Bashir F. H., Othman M. S., Mazlan A. G., Rahim S. M., Simon K. D. (2013). Heavy metal concentration in fishes from the coastal waters of Kapar and Mersing, Malaysia. *Turkish Journal of Fisheries and Aquatic Sciences*.

[11] Jolly Y. N., Islam A., Akbar S. (2013). Transfer of metals from soil to vegetables and its possible health risk assessment. *SpringerPlus*.

[12] Islam A., Jolly Y. N. (2007). Heavy metals in water and fishes of the tannery affected vicinity of the River Buriganga. *Journal of Bangladesh Academy of Sciences*.

[13] Jolly Y. N., Chowdhury T. R., Islam A., Suravi N. I., Sultana M. S. (2012). Background chemical study of relocated hazaribagh tannery complex environment, Savar. *Journal of Bangladesh Academy of Sciences*.

[14] AOAC (2000). *Association of Official Analytical Chemists*.

[15] WHO/FAO (1984). *List of Maximum Levels Recommended for Contaminants by the Joint FAO/WHO*.

[16] Sen I., Shandil A., Shrivastava V. S. (2011). Study for determination of heavy metals in fish species of the river Yamuna (Delhi) by inductively coupled plasma-optical emission spectroscopy (ICP-OES. *Advances in Applied Science Research*.

[17] Ganbi H. H. A. (2010). Heavy metals pollution level in marine hammour fish and the effect of popular cooking methods and freezing process on these pollutants. *World Journal of Dairy & Food Sciences*.

[18] Imtiazuddin S. M., Mumtaz M. (2013). Toxicity study of heavy metals textile pollutants in wastewater effluent on the fishes of Karachi harbour area. *WebPub*.

[19] Marimuthu K., Thilaga M., Kathiresan S., Xavier R., Mas R. H. M. H. (2012). Effect of different cooking methods on proximate and mineral composition of striped snakehead fish (*Channa striatus,* Bloch). *Journal of Food Science and Technology*.

[20] Kumar B., Sajwan K. S., Mukherjee D. P. (2012). Distribution of heavy metals in valuable coastal fishes from North East Coast of India. *Turkish Journal of Fisheries and Aquatic Sciences*.

[21] Das M., Das R. (2012). Need of education and awareness towards zinc supplementation: a review. *International Journal of Nutrition and Metabolism*.

[22] Swami K., Judd C. D., Orsini J., Yang K. X., Husain L. (2001). Microwave assisted digestion of atmospheric aerosol samples followed by inductively coupled plasma mass spectrometry determination of trace elements. *Analytical and Bioanalytical Chemistry*.

[23] Gokoglu N., Yerlikaya P., Cengiz E. (2004). Effects of cooking methods on the proximate composition and mineral contents of rainbow trout (*Oncorhynchus mykiss*). *Food Chemistry*.

[24] Mukherjee D. P., Bhupander K. (2011). Assessment of arsenic, cadmium and mercury level in commonly consumed coastal fishes from Bay of Bengal, India. *Food Science and Quality Management*.

[25] Rejomon G., Nair M., Joseph T. (2010). Trace metal dynamics in fishes from the southwest coast of India. *Environmental Monitoring and Assessment*.

[26] World Health Organization (1989). Heavy metals-environmental aspects. *Environmental Health Criteria*.

[27] Bernard A. (2008). Cadmium & its adverse effects on human health. *Indian Journal of Medical Research*.

[28] Satarug S., Moore M. R. (2004). Adverse health effects of chronic exposure to low-level cadmium in foodstuffs and cigarette smoke. *Environmental Health Perspectives*.

[29] WHO (World health organization) (1985). *Guidelines for Drinking Water Quality. Recommendation*.

[30] Abhijit M., Shreya M., Sumit H., Anish C. (2000). Heavy metal concentrations in India coastal fishes. *Research Journal of Chemistry and Environment*.

[31] Department of Water Affairs and Forestry (1996). *South African Water Quality Guidelines, Aquatic Ecosystems*.

[32] Lokesshappa B., Kandarp S., Vivek T., Anil K. D. (2012). Assessment of toxic metals in agricultural produce. *Food and Public Health*.

[33] NAS/NRC (National Academy of Sciences/National research Council) (1997). *Arsenic in Drinking Water*.

[34] FAO/ WHO (2004). *Summary of Evaluations Performed by the Joint FAO/WHO Expert Committee on Food Additives (JECFA 1956-2003)*.

[35] FAO/WHO food standards programme

[36] USEPA (2000). Guidance for assessing chemical contaminant, data for use in fish advisories. Fish sampling and Analysis. *EPA*.

[37] Datar M. D., Vashishtha R. P. (1990). Investigation of heavy metals in water and silt sediments of Betwa River. *Indian Journal of Environmental Protection*.

[38] Asghari L., Zeynali F., Sahari M. A. (2013). Effects of boiling, deep-frying, and microwave treatment on the proximate composition of rainbow trout fillets: changes in fatty acids, total protein, and minerals. *Journal of Applied Ichthyology*.

[39] Oluwaniyi O. O., Dosumu O. O. (2009). Preliminary Studies on the effect of processing methods on the quality of three commonly consumed marine fishes in Nigeria. *Biokemistri*.

[40] Alam C. M. K. (2004). *Bangladesh's Maritime Challenges in the 21st Century*.

[41] UNEP (1986). Environmental problems of the marine and coastal area of Bangladesh. *National Report UNEP Regional Seas Reports and Studies No.*.

[42] Hossain M. M. National Report of Bangladesh on coastal pollution loading and water quality criteria of the Bay of Bengal large marine ecosystem (BOBLME) (GCP/RAS/236/GEF).

